# MicroRNA-183/96/182 cluster in immunity and autoimmunity

**DOI:** 10.3389/fimmu.2023.1134634

**Published:** 2023-02-20

**Authors:** Zhuang Wang, Rujuan Dai, Sattar Ansar Ahmed

**Affiliations:** Department of Biomedical Sciences and Pathobiology, Virginia-Maryland College of Veterinary Medicine (VMCVM), Virginia Tech, Blacksburg, VA, United States

**Keywords:** microRNA, miR-183/96/182 cluster, immunity, autoimmune, autoinflammatory diseases, epigenetic regulation

## Abstract

MicroRNAs (miRNAs) are crucial post-transcriptional regulators of gene expression in ubiquitous biological processes, including immune-related pathways. This review focuses on the miR-183/96/182 cluster (miR-183C), which contains three miRNAs, miR-183, -96, and -182, having almost identical seed sequences with minor differences. The similarity among seed sequences allows these three miRNAs to act cooperatively. In addition, their minor differences permit them to target distinct genes and regulate unique pathways. The expression of miR-183C was initially identified in sensory organs. Subsequently, abnormal expression of miR-183C miRNAs in various cancers and autoimmune diseases has been reported, implying their potential role in human diseases. The regulatory effects of miR-183C miRNAs on the differentiation and function of both innate and adaptive immune cells have now been documented. In this review, we have discussed the complex role of miR-183C in the immune cells in both normal and autoimmune backgrounds. We highlighted the dysregulation of miR-183C miRNAs in several autoimmune diseases, including systemic lupus erythematosus (SLE), multiple sclerosis (MS), and ocular autoimmune disorders, and discussed the potential for utilizing miR-183C as biomarkers and therapeutic targets of specific autoimmune diseases.

## 1 Introduction

MicroRNAs (miRNAs) are small non-coding RNAs (mostly 20~24 nucleotides) that regulate gene expression at the post-transcriptional level (1, 2). Since the discovery of the first microRNA (miRNA), *lin-4*, in *C. elegans* by Lee et al. and Wightman et al., a large number of miRNAs have been identified in plants, animals, bacteria, and viruses, implying that miRNAs are conserved across species (3–8). Approximately 48,860 mature microRNAs in 271 organisms have been recorded on the miRbase database released in 2019, and it is predicted that this number will increase in the future (8). miRNAs have been shown to epigenetically regulate the expression of target mRNAs by binding to complementary sequences. Given that each miRNA targets many mRNAs, almost all cellular pathways are predicted to be subjected to miRNA-mediated regulation (1, 9–11). Various miRNAs, individually or cooperatively, regulate the normal development and function of biological systems in animals (7, 12–15). Abnormal expression of miRNAs in multiple human diseases, including cancers and autoimmune diseases, have been reported, implying a conceivable role of miRNAs in human disease pathogenesis. The specific disease-related miRNAs could serve as diagnostic biomarkers and potential therapeutic targets of the disease (16–19).

The microRNA-183/96/182 cluster (miR-183C) is a highly conserved microRNA cluster containing three paralogous microRNAs (miR-183, miR-96, and miR-182), located on human chr7q32.2 and mouse chr6qA3 (20). The first identified miRNA of human miR-183C is miR-96 in human cancer HELA cells (21). Subsequently, miR-182 and miR-183 in humans were identified by different groups as a cluster (22, 23). miR-96 was later classified as a member of miR-183C due to their sequence similarity, co-expression, and close chromosomal location (20). The miR-183C miRNAs are markedly expressed in the sensory organs (such as eye and ear) and play an essential role in the normal development and function of sensory organs (20, 24). Interestingly, although miR-183C miRNAs are expressed at a low level in non-sensory organs, the upregulation of miR-183C miRNAs has been noticed in various cancers (such as prostate cancer, breast cancer, lung cancer, melanoma), and in autoimmune and inflammatory diseases (such as systemic lupus erythematosus or SLE and multiple sclerosis or MS) (25–31). The crucial role of miR-183C miRNAs in the immune system and autoimmune diseases has been reported in recent studies (32–35). In this review, we summarized previous research on miR-183C involvement in immunity and autoimmunity and discussed the current understanding of the regulatory effect of miR-183C on immune cell functions and autoimmune disease pathogenesis.

## 2 Expression and function of miR-183C in sensory organ

The expression of the polycistronic miR-183C microRNAs starts from the transcription of a long single hairpin-shaped pri-miR/183/96/182 transcript that contains three miRNAs with similar seed sequences (20, 31, 35). The miR-183C is highly conserved in various species, and the miR-183C is expressed at high levels in sensory organs, such as the retina, olfactory epithelia, and inner ear (20, 36). The crucial role of miR-183C miRNAs in sensory organs development and function has been reported by different groups (37–42).

The mutations of the miR-96 seed region are responsible for progressive hearing loss in both humans and mice (37, 38). The *Mir96^Dmdo^* mice, which carry an *N*-ethyl-*N*-nitrosurea (ENU)-induced A to T mutation in the seed sequence of miR-96, had degeneration of hair cells in homozygotes by seven days and the loss of any detectable cochlear nerve activity by four weeks of age (37). By mapping the genes of an autosomal dominant deafness locus, Mencia et al. determined that the mutations at the human miR-96 seed region caused autosomal dominant progressive hearing loss in humans (38). Furthermore, a group of deafness-related genes was identified in the miR-96-mediated gene regulatory network, suggesting a vital role of miR-96 in inner ear development and progressive deafness (39). Further studies have shown that the mice with the inactivation of miR-183/96/182 cluster (miR-183C^GT/GT^) or deletion of both miR-96 and miR-183 (*miR-183/96^dko^*) had congenital deafness with severe defects in hair cell development and function (40, 41). The miR-182 single knockout (*miR-182^ko^*) mice only exhibited progressive hearing loss (40). The expression of the three miR-183C miRNAs was progressively increased during mouse retinal development, and their expression was highly induced by the light (20, 43). Inactivation of the miR-183/96/182 cluster resulted in retinal dysfunction and degeneration with progressive electroretinogram defects and increased sensitivity to light damage in miR-183C^GT/GT^ mice (42). Although deleting miR-182 in B6 mice did not induce any apparent histological changes in the retina, it led to the decline of retinal function (44, 45). Together, the above studies strongly demonstrated the essential role of miR-183C miRNAs, either individually or collectively, in the development and function of the retina and inner ear.

## 3 Expression and function of miR-183C in immune cells

**Figure 1:**
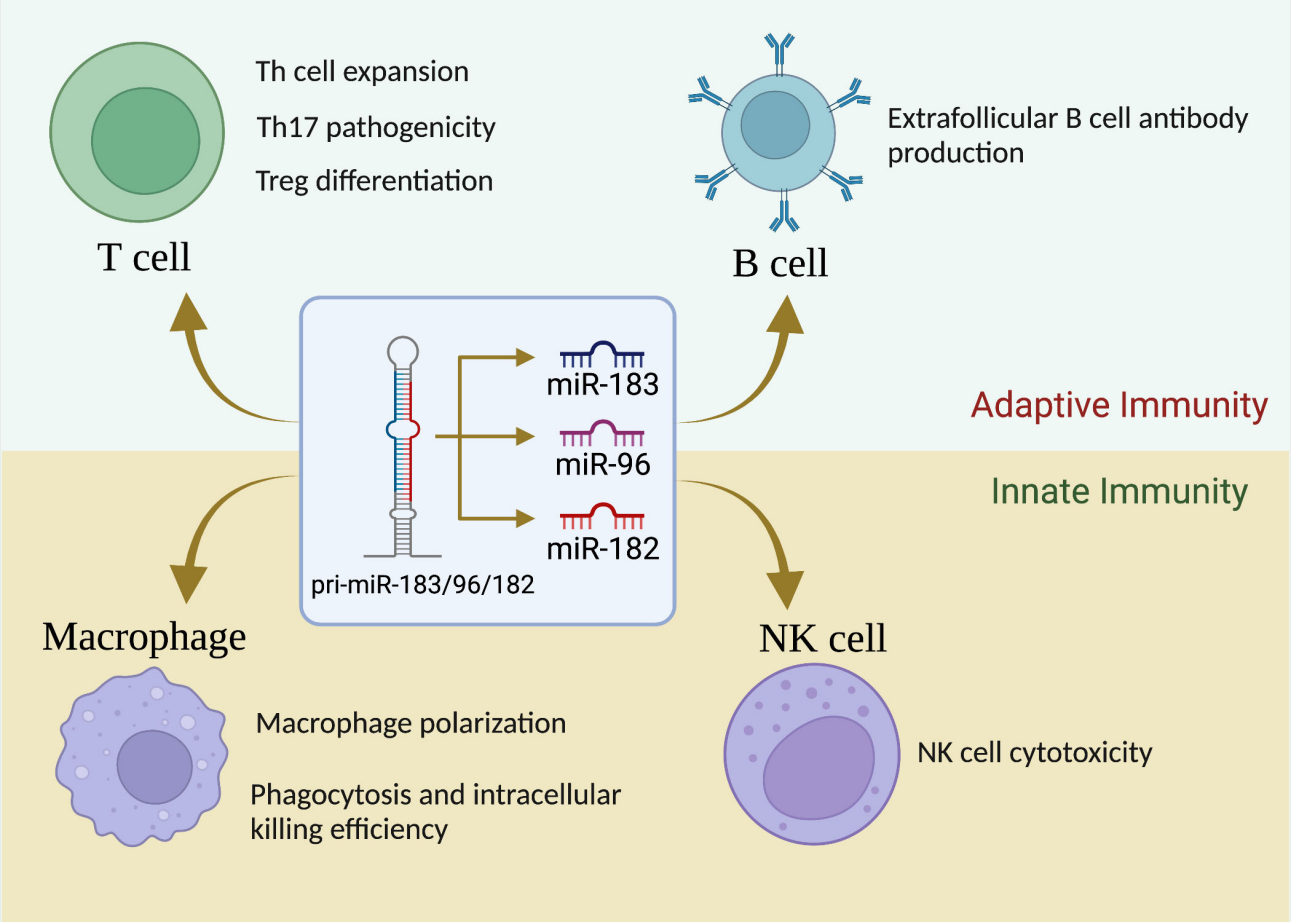
miR-183C is a crucial regulator of immune cell development, differentiation and function. While miR-183C miRNAs are dispensable for T-cell development, they play a vital role in Th1/Th17/Treg differentiation, proliferation, and function. miR-183C miRNAs have minimal effect on B-cell development, GC response, and antibody production, although miR-182 appears to have a promoting effect on extrafollicular B-cell response and antibody production. In addition, miR-183C miRNAs play an important role in regulating macrophage polarization, phagocytosis, and innate NK cell cytotoxicity.

Unlike in the sensory organs, miR-183C miRNAs expression was not detectable in unstimulated cells of the immune system (basal state) in normal mice (20). However, upon stimulation with different antigens and cytokines, the expression of miR-183C was highly upregulated in both adaptive and innate immune cells (33, 46). **Figure 1** summarizes the current understanding of the role of miR-183C on the development and functions of different subpopulations of the cells of the immune system.

### 3.1 T cells

**Figure 2:**
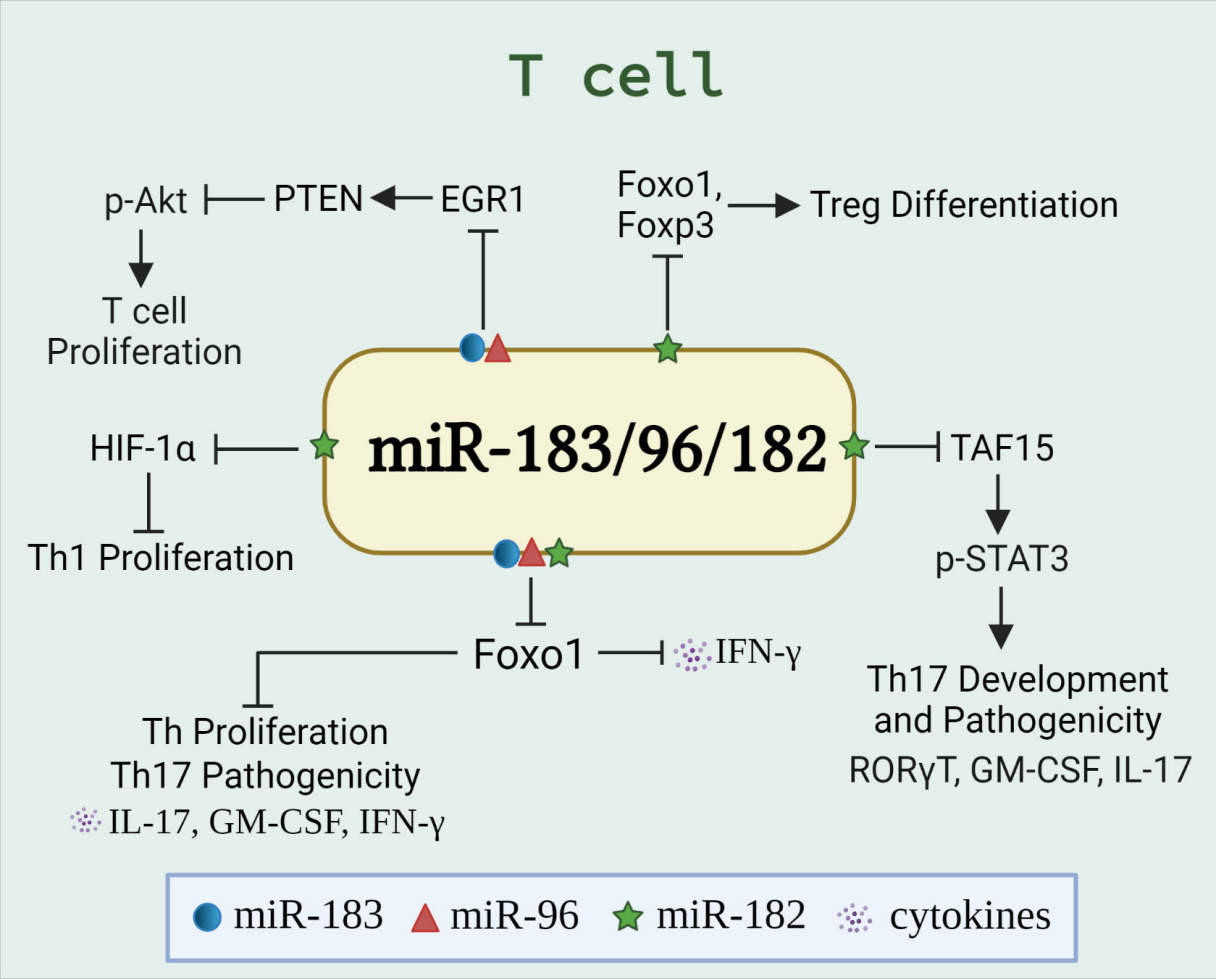
miR-183C regulates the differentiation, proliferation, and function of T cells. The schematic graph illustrates the miR-183/96/182-regulated genes and pathways, either individually or collectively, in T cells. The miR-183C promotes T cell proliferation and IFNγ production *via* targeting Foxo1 (32, 34), HIF-1α (27), or EGR1/PTEN/Akt pathway (49). In addition, miR-182 suppresses Treg differentiation by targeting Foxo1 and Foxp3 (50). While miR-183C has been shown to promote the pathogenicity of Th17 cells by inhibiting Foxo1 (33), miR-182 has been shown to inhibit the Th17 development and pathogenic response *via* targeting TAF15/STAT3 signaling pathway (51).

T lymphocytes (T cells) coordinate the activities in other white blood cells, either *via* secreting cytokines or by direct cell-to-cell contact. T cells are indispensable for the effective countering of intracellular infections, especially by secreting proinflammatory cytokines [interferon-gamma (IFNγ) or interleukin-17 (IL-17)] and inducing cytotoxicity of infected cells. Dysregulated T cell numbers and/or functions are evident in many autoimmune diseases (47, 48). These include enhanced pathogenicity of T helper 17 (Th17) cells or impaired function of regulatory T (Treg) cells (which normally hold-in-check autoreactive cells), or increased secretion of proinflammatory cytokines (such as IL-17 and IFNγ). Of note, the expansion and function of Th1/Th17/Treg cells were found to be regulated by miR-183C miRNAs *via* targeting different genes/signaling pathways in different experimental settings (**Figure 2**), suggesting a crucial role of miR-183C miRNAs in T-cell immunity and autoimmunity.

miR-182 expression was highly upregulated in the activated Th cell subsets (Th1, Th2, and Th17) to promote Th cell expansion *via* targeting Foxo1 (32). Th1 and Th17 are proinflammatory immune cells that are thought to promote autoimmune diseases (52, 53). Inhibition of miR-182 with antagomir-182 significantly reduced Th cell expansion *in vitro* and *in vivo* due to the lower proliferation and higher cell death rates (32). In a murine model of ovalbumin (OVA)-induced arthritis, miR-182 has been shown to promote inflammation. The RAG1^-/-^BALB/c mice receiving antagomir-182 transfected Th cells had reduced OVA-induced arthritis with amelioration of knee swelling and significantly reduced inflammation and tissue destruction (32). MiR-182 was also found to be upregulated in peripheral CD4^+^ T cells from multiple sclerosis patients, which was associated with augmented production of proinflammatory cytokine IFNγ and IL-17 (27). In experimental autoimmune encephalomyelitis (EAE) mice, overexpression of miR-182 promoted the expansion of IFNγ- and IL-17- expressing T cells *in vivo* and exacerbated clinical score. Further *in vitro* experiments suggested that miR-182 promoted the differentiation of inflammatory T cells, especially Th1 cells *via* targeting hypoxia-inducible factor-1α (HIF-1α), a regulator of inflammation (27).

miR-183 and miR-96, akin to miR-182 also promote T cell proliferation. Both miR-96 and miR-183 were shown to regulate CD4^+^CD25^-^ T cell proliferation *via* the EGR1/PTEN/Akt pathway (49). Overexpression of miR-183 and miR-96 in CD4^+^ T cells reduced Early growth response 1 (EGR1) expression directly, which led to reduced expression of Phosphatase and tensin homolog (PTEN) expression and consequently increased Protein kinase B (PKB or Akt) phosphorylation and T cell proliferation. Adoptively transferring of miR-183 and miR-96 overexpressing hemagglutinin (HA)-specific CD4^+^CD25^-^ T cells into autoimmune diabetes-prone INS-HA/Rag2KO mice resulted in the expansion of antigen-specific CD4^+^ T cells and accelerated the development of autoimmune diabetes (49). Further, simultaneous inhibition of miR-183 and miR-96 in CD4^+^CD25^-^ T cells with specific antagomirs promoted EGR1 and PTEN expression and suppressed T cell proliferation. Adoptively transferring antagomir-183/96 transfected HA-specific CD4^+^CD25^-^ T cells into INS-HA/Rag2KO mice delayed the onset of autoimmune diabetes (49).

miR-183C miRNAs were highly induced in differentiated Th17 cells, particularly in pathogenic Th17 cells through the activation of the IL-6/STAT3 pathway (33). Notably, these investigators demonstrated that miR-183C miRNAs are essential for the pathogenic function of Th17 cells by positively regulating cytokines involved in the pathogenicity of Th17 cells without affecting the differentiation of this subset. Overexpression of miR-183C miRNAs, especially miR-96, in primary CD4^+^ T cells, promoted the production of inflammatory cytokines IL-17A, IL-17F, GM-CSF, IL-22, and IFNγ in differentiated pathogenic Th17 cells. In *miR-183C*-deficient pathogenic Th17 cells, the expression of these inflammatory cytokines was significantly depressed compared to *miR-183C*-sufficient controls (33). The clinical score of myelin oligodendrocyte glycoperotein_35-55_ (MOG_35-55_)-induced EAE in the Rag1^-/-^ mice that received CD4^+^ T cells from miR-183C^-/-^ knockout mice was significantly reduced compared to the Rag1^-/-^ mice receiving CD4^+^ T cells from control miR-183C^+/+^ mice. Mechanistically, the authors demonstrated that the miR-183C miRNAs positively regulated the pathogenic function of Th17 cells partially by inhibiting the expression of Foxo1, a negative regulator of Th17 cell pathogenicity (33).

The above study demonstrated that all three miR-183C miRNAs had a promoting effect on the pathogenic function of Th17 cells derived from normal B6 mice without affecting the Th17 cell differentiation, with miR-96 being the most potent (33). However, in a mouse model of autoimmune disease experimental autoimmune uveitis (54), miR-182 had an inhibitory effect specifically on the Th17 cell development and pathogenic function (51). Zhang et al. reported that miR-182 was downregulated in the EAU mouse-derived Th17 cells. Inhibition of miR-182 significantly increased the expression of Th17 signature genes such as *IL-17*, *IL-22*, *GM-CSF*, and *RORγT* in EAU mice-derived Th17 cells. Mechanistically, miR-182 inhibited Th17 cell development by directly targeting and inhibiting the transcriptional initiator TATA-binding protein-associated factor 15 (TAF15). *In vitro* studies indicated that the overexpression of TAF15 was able to rescue the miR-182 overexpression-induced reduction of signal transducer and activator of transcription 3 (STAT3) phosphorylation, an important upstream regulator of Th17 (51, 55).

In several human autoimmune diseases, the proportion of proinflammatory Th17 cells was increased, while the immunotolerant Treg cell differentiation was suppressed. In contrast to its promoting role on Th17 cell differentiation, miR-182 plays a suppressing role in CD4^+^CD25^+^Foxp3^+^ Treg differentiation. Both *in vitro* and *in vivo* studies demonstrated that the knockdown of miR-182 with a lentiviral vector expressing miR-182 inhibitor (anti-LV-miRNA182) promoted the differentiation of Tregs *via* upregulating the expression of *Foxo1* and *Foxp3*. The miR-182 knockdown mice were more resistant to MOG_35-55_-induced EAE disease than control mice due to increased differentiation of CD4^+^CD25^+^Foxp3^+^ Tregs in peripheral lymphoid organs of the miR-182 knockdown mice (50). However, an *in vitro* study demonstrated that overexpression of miR-182 in Jurkat T cells, a CD4^+^ cell line derived from human acute T cell leukemia, downregulated Foxo1 expression and promoted the polarization of the transduced T cells to Foxp3^+^ T cells, of which some were Foxp3^+^IL-17^+^ cells (56). The above studies indicated that the miR-182 could have either a positive or negative role in regulating Tregs/Th17 differentiation, depending on the pathological conditions, experimental settings, and types of cells.

In addition, the expression of miR-183C miRNAs was dynamically regulated in discrete phases of invariant natural killer T (iNKT) cell maturation. The expression of miR-183C miRNAs is crucial for the development, maturation, and function of iNKT cells (57). The miR-183C^-/-^ knockout mice showed a reduced proportion and absolute numbers of iNKT cells in the thymus and spleen, depressed terminal maturation of thymic iNKT cells. Meanwhile, the percentages of IL-17-producing NKT cells (NKT17) out of both thymic and splenic iNKT cells were dampened in miR-183C^-/-^ mice compared with wild-type mice (57). Mechanistically, Foxo1, Foxo3, EGR1, and EGR2 were the potential targets of miR-183C in iNKT cells. miR-183C miRNAs are thought to promote NKT17 effector function through inhibiting Foxo1 (57).

While miR-182 is highly upregulated in activated T lymphocytes, surprisingly, the systemic knockout of miR-182 in B6 mice had no obvious effect on the development and function of the T cell subsets such as CD4^+^, CD8^+^, CD4^-^CD8^-^ double negative (DN) T cells, and Tregs (58). Furthermore, the T cell-dependent immune response to *Listeria monocytogenes* in miR-182^-/-^ mice was similar to that in the controls (58). The dispensable role of miR-182 in lymphocyte development and function of B6 mice is likely due to the compensation by miR-96 and miR-183 since the three miRNAs have similar seed sequences and overlaps of the target genes.

In summary, although the molecular mechanism needs to be further clarified, the current studies demonstrated an important role of miR-183C miRNAs in the regulation of Th cell expansion, Th17/Treg differentiation and function, and T cell-mediated inflammation in specific pathological conditions.

### 3.2 B cells

The critical role of miRNAs on B cell development and activation has been extensively discussed (59–61). Similar to T cells, the miR-183C is expressed at a very low level in naïve B cells and is markedly upregulated in activated B cells (58, 62). However, compared with T cells, there are fewer reports of the role of miR-183C miRNAs in B cells. The dispensable role of miR-182 and miR-183C miRNAs on B cell development and differentiation has been consistently documented in different studies (58, 62, 63). In miR-182^-/-^ and miR-183C^-/-^ mice, the proportions of B cells at different developmental stages in the bone marrow and differentiation stages in the spleens mainly remained unchanged when compared to wild-type control mice (58, 62, 63).

Nevertheless, inconsistent results were obtained with regard to the role of miR-182 on B cell response and function. Pucella et al. reported that miR-182^-/-^ mice have a similar B cell response to the immunization with T cell-dependent (TD) antigen (NP_(30)_-CGG) as the control B6 mice with similar fractions of germinal center (GC) B cells and normal production of both low-affinity and high-affinity NP-specific IgG1 at 21 days post-immunization (58). On the other hand, Li et al. reported that the production of NP-specific IgM and IgG1 was inhibited at early stages (7- and 14-days post TD antigen NP_38_-CGG immunization) in miR-182^-/-^ mice, although the formation of germinal center B cells (GC B) and the differentiation of follicular helper T cells (Tfh) were not compromised in miR-182^-/-^ B6 mice (62). In miR-182^-/-^ mice, the fraction of NP-specific IgG1-secreting cells in the spleen was reduced compared to controls at five days post-immunization with NP_38_-CGG but remained at a similar level at ten days (62). Immunization of mice with NP_25_-Ficoll, a T cell-independent type II (TI II) antigen, confirmed that miR-182 deficiency affected extrafollicular plasma B cell generation. The miR-182^-/-^ mice showed a more general reduction in various subtypes of anti-NP_25_-Ficoll antibodies throughout all time points (4-, 7-, 14-, and 21-days post-immunization) and a reduction in IgG1 secreting cells in the spleen at day five (62). Therefore, these authors concluded that miR-182 plays an important role in driving extrafollicular B-cell response to T cell-dependent (NP_38_-CGG) and independent (NP_25_-Ficoll) antigens without affecting GC responses.

Considering the potential compensation effect of miR-96 and miR-183 on the deletion of miR-182 individually, Pucella et al. utilized a whole miR-183C cluster knockout (miR-183C^GT/GT^) mouse model to investigate the effect of miR-183C on the humoral response to antigens (63). In this study, these workers further showed that miR-183C miRNAs were dispensable for B cell development and function. The miR-183C^GT/GT^ mice had normal GC response to the immunization of TD antigens, either sheep red blood cell (SRBC) or NP-CGG, although there was a mild light zone GC skew in miR-183C^GT/GT^ mice. In miR-183C^GT/GT^ mice, NP_(31)_-CGG immunization induced comparable levels of serum total IgM and IgG1 and serum NP-specific IgM as wild-type littermates at different time points, while a slight but significant reduction of NP-specific serum IgG1 was observed in 28 days after immunization. Further, the authors demonstrated that miR-183C miRNAs were not required for the extrafollicular humoral responses to both types I and II TI antigens at either early or late stages of immunization. There was no significant difference in the proportion of plasmablasts (CD138^+^B220^lo^) and intracellular IgM^+^ cells observed in TI-1 antigen LPS immunized miR-183C^GT/GT^ and miR-182^-/-^ mice compared to their respective control mice. Similarly, there were equivalent frequencies of IgM, IgG1, and IgG3 secreting plasmablasts and NP-specific IgM and IgG secreting cells in TI antigen NP-Ficoll immunized miR-183C^GT/GT^ and miR-182^-/-^ compared to control mice (63). Thus, Pucella et al. concluded that miR-183C miRNAs had a minimal role in primary B-cell antibody response to either TD or TI antigens, which are predominately involved in GC and extrafollicular B-cell response, respectively.

Together, current studies indicated that miR-183C miRNAs are largely dispensable for B cell development and GC B-cell response (62, 63). The reasons for the discrepancies between the two studies about the role of miR-182 on extrafollicular B-cell responses remain unclear, although it might be explained by the difference in NP-specific IgG1 antibody-secreting plasmablasts in control mice (63).

### 3.3 Macrophages

**Figure 3:**
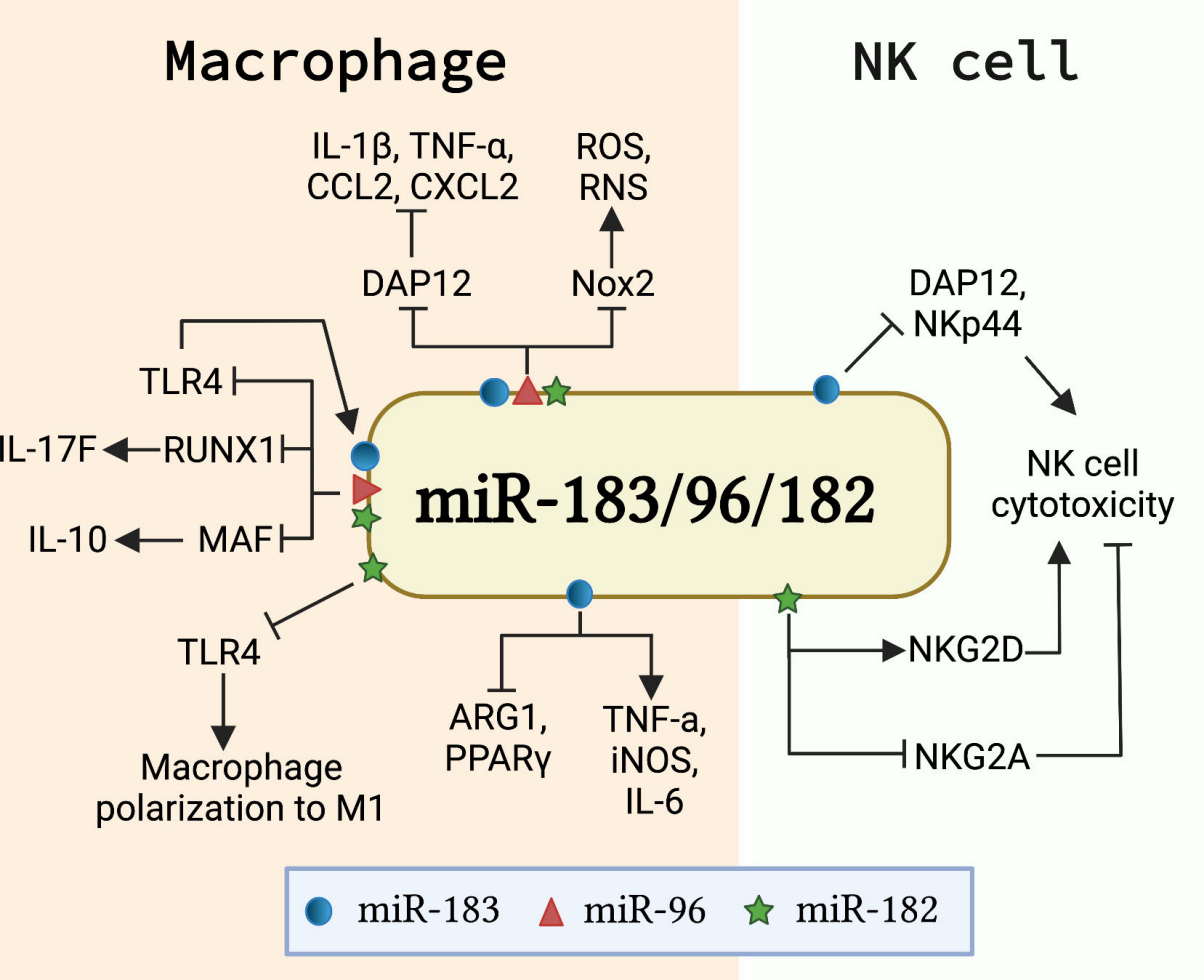
miR-183C is involved in the development and function of innate immune cells. The schematic graph illustrates the major genes/signaling pathways that mediate the function of miR-183C in macrophages and NK cells. In macrophages, miR-183C regulates the expression of IL-10 and IL-17F by targeting MAF and RUNX1, respectively, and provides a negative feedback regulation of TLR4 signaling (68). miR-183C also regulates the inflammatory responses and phagocytosis function in macrophages by inhibiting DAP12 and Nox2, respectively (69). In addition, miR-182 and miR-183 promote M1 macrophage polarization *via* different mechanisms. miR-182 targets TLR4 to regulate the macrophage polarization (70). miR-183 promotes M1 signature genes (TNFα, iNOS, IL-6) expression and inhibits M2 macrophage signature genes (ARG1 and PPARγ) expression (71). In NK cells, miR-182 and miR-183 have opposite effects on the cytotoxicity of NK cells. While miR-182 promotes cytotoxicity by suppressing the inhibitory receptor NKG2A and increasing activating receptor NKG2D expression (72), miR-183 inhibits the cytotoxicity of NK cells by targeting DAP12 and NKp44 (46, 73).

Macrophages are potent phagocytotic cells essential for the removal of aging and dying apoptotic cells. It is well-accepted that impaired phagocytosis of macrophages in autoimmune disease patients results in inadequate clearance of dead cell debris. Consequently, the accumulation of apoptotic debris provides a source of autoantigens that potentially contributes to the initiation of autoimmune diseases (64, 65). Macrophages also contribute to the development of autoimmunity through producing inflammatory cytokines/chemokines or shifting the macrophage polarization (66, 67). Recent *in vivo* and *in vitro* studies showed that miR-183C miRNAs regulate macrophage functions *via* targeting different genes and pathways (**Figure 3**). miR-183C regulated the number of CD11b^+^F4/80^+^ corneal resident macrophages and inhibited IL-17 and IL-10 production in corneal resident macrophages through targeting *Runx1* and *Maf* (68). The inactivation of miR-183C in miR-183C^GT/GT^ mice led to increased corneal resident macrophages number and enhanced IL-17 and IL-10 production in corneal resident macrophages following *Pseudomonas aeruginosa* (PA) infection (68). Also, miR-183C provided a negative feedback regulation on TLR4 signaling pathway (68). Another study reported that PA or LPS treatment significantly induced miR-183 and miR-182, but not miR-96, in macrophage cell line RAW264.7 cells (69). Inhibition of miR-183C miRNAs in RAW264.7 cells significantly inhibited the inflammatory response to either LPS or PA treatment with reduced production of CCL2, CXCL2, and/or TNF-α when compared to control cells. The peritoneal macrophages from miR-183C^GT/GT^ mice had higher basal levels of proinflammatory cytokines IL-1β, CCL2, and CXCL2 than controls, but had reduced responsiveness to *in vitro* treatment with LPS or PA (69). The authors suggested that miR-183C potentially inhibited DNAX-activating protein of 12 kDa (DAP12), a negative regulator of TLRs responses, to regulate inflammatory responses in macrophages (69). In addition to regulating cytokine/chemokine production in macrophages, miR-183C also regulates the phagocytosis function of macrophages. A bioinformatics analysis approach revealed that the predicted target sites of miR-182 were over-presented in a set of macrophage intracellular pathogen response (macIPR) genes (74). Further, Gregory et al. demonstrated that overexpression of miR-182 in primary human alveolar macrophage-like monocyte-derived macrophages (MDM) increased proinflammatory cytokines genes expression and enhanced autophagy function of macrophages during infection with *F. tularensis* live vaccine strain (LVS) (74). Overall, this study showed an enhancing effect of miR-182 on the autophagy function of macrophages against infection. However, the suppressing effect of miR-183C miRNAs on phagocytosis has also been reported (75). Inactivating miR-183C significantly reduced the severity of PA-induced keratitis in miR-183C^GT/GT^ with increased efficiency for bacterial cleaning. Overexpression of miR-183C miRNAs significantly inhibited phagocytosis function and intracellular killing efficiency in mouse macrophage cell line RAW264.7, while inhibition of miR-183C miRNAs increased phagocytosis function and intracellular killing efficiency. The increased phagocytosis capability and intracellular killing efficiency were also observed in the polymorphonuclear neutrophils (PMNs) from miR-183C^GT/GT^ mice compared to the cells from the control mice (75). In a later study by the same group, the authors demonstrated that miR-183C limited the phagocytosis intracellular killing capacity of macrophages *via* targeting NADPH oxidase 2 (Nox2) to suppress the production of reactive nitrogen (RNS) and oxygen species (ROS), two key players in macrophage antimicrobial activity (69). Moreover, miR-183C miRNAs have been shown to regulate the polarization of macrophages. Inhibition of miR-183 in bone marrow-derived macrophages (BMDMs) dramatically decreased M1 macrophage-associated genes (*TNF-α, iNOS, IL-6*) expression in response to oxidized low-density lipoprotein (Ox-LDL) stimulation, while concomitantly increasing M2 macrophage-associated genes (*Arg1, TGF-β, PPARγ*) expression (71). Furthermore, miR-182 has been shown to be a major player in mesenchymal stem cells-derived exosomes (MSC-Exo)-mediated attenuation of myocardial ischemia/reperfusion (I/R) injury by modulating macrophage polarization from inflammatory M1 macrophage toward M2 (70). RAW264.7 macrophages receiving miR-182 inhibitor transfected MSC-Exo had reduced expression of M1 markers and increased expression of M2 markers compared to the cells receiving negative control inhibitor transfected MSC-Exo. Mechanistically, miR-182 regulates macrophage polarization by targeting TLR4 to influence the crosstalk between TLR4/NF-κB pathway and PI3K/Akt pathway (70).

### 3.4 NK cells

Nature killer (NK) cells are innate lymphocytes that play an important role in killing tumor cells and virus-infected cells (76, 77). Transforming growth factor β1 (TGF-β) has been shown to suppress natural killer (NK) cell function by inducing miR-183 to inhibit the expression of DAP12, a critical regulator of NK cell effector response (46, 78). Overexpression of miR-183 significantly suppressed DAP12 and surface receptor NKp44 expression in primary NK cells and NK cell line NK92 cells, which led to reduced cytotoxicity of NK92 cells (46). A separate study confirmed that TGF-β induced miR-183 expression in NK cells suppressed DAP12 (73). In this study, the researchers demonstrated that platelet-derived ectosomes (PLT-Ecto) transfused NK cells had a suppressed level of intracellular IFNγ and DAP12 *via* TGF-β mediated induction of miR-183 (73). Overall, the above two studies showed that miR-183 suppresses NK cell function by inhibiting the expression of DAP12. In addition, increased expression of miR-182 was found in NK cells of hepatocellular carcinoma patients (72). Overexpression of miR-182 in NK cells increased the expression level of activating receptor NKG2D and suppressed the inhibitory receptor NKG2A, leading to an enhanced NK cells cytotoxicity against tumorigenic Huh-7 cells (72). Together, these works suggested an opposite role of miR-182 and miR-183 in regulating NK cell function, potentially through the regulation of different genes and pathways (**Figure 3**).

## 4 miR-183C miRNAs in autoimmune disease and potential therapeutic application

The dysregulated expression of miR-183C miRNAs among different cell types has been found in various autoimmune diseases such as SLE, MS, RA, EAU, graves’ orbitopathy, and sympathetic ophthalmia with uveitis and implicated in disease pathogenesis (**Table 1**). Given their altered expression in autoimmune diseases and crucial immune regulatory roles, miR-183C miRNAs have been considered as potential therapeutic targets for immune-related diseases in several studies. In the following section, we focus on the dysregulation and the potential for therapeutic intervention of miR-183C miRNAs in SLE, MS, and autoimmune-mediated eye diseases.

**Table 1:** Dysregulation of miR-183C in autoimmune diseases.

microRNA	Expression	Cell/tissues	Species/Strains	Disease	Potential pathogenic contribution	References
miR-183, miR-96, miR-182	Up	Splenocytes, PBMCs, CD4^+^T	MRL/lpr, B6/lpr, NZB/W_F1,_ C3.MRL-Fas^lpr^	SLE	Promoted inflammatory cytokine IFNγ and disease *via* targeting Foxo1	(28, 34, 79–81)
miR-182, miR-183	Up	PBMC	Human	SLE	Not assessed (N/A)	(82, 83)
miR-182	Up	kidney tissue	MRL/lpr	SLE/LN	Promoted renal fibrosis and LN development *via* targeting Foxo1	(81)
miR-183	Down	kidney tissue	MRL/lpr, human	SLE/LN	Promoted renal damage by blunting the inhibitory effect of miR-183 on mTOR or TGF-β/Smad/TLR3 signaling pathway.	(84, 85)
miR-183	Down	Plasma	Human	SLE/LN	N/A	(86)
miR-182	Up	CD4^+^ T	Human, C57BL/6 mouse	RRMS, EAE	Enhanced Th1 expansion, IFNγ and IL-17 production *via* targeting HIF-1α; Suppressed Treg differentiation *via* targeting Foxo1 and Foxp3	(27, 50)
miR-96	Up	PBMC	Human	Remission MS	N/A	(29)
miR-96	Down	Plasma	Human	RA	N/A	(87)
miR-183-3p	Down	Psoriatic lesioned skin	Human	Psoriasis	Promoted proliferation and migration of keratinocytes by increasing miR-183 target gene, GAB1.	(88)
miR-96, miR-183	Up	CD4^+^ T	Human	Graves’ orbitopathy	Promoted T cell proliferation and activation *via* regulating EGR1/PTEN/AKT pathway	(49)
miR-182, miR-183	Up	Ocular tissue	Rat	EAAU	N/A	(89)
miR-182, miR-183	Down	Ocular tissue	Human	sympathetic ophthalmia	N/A	(90)
miR-182	Down	CD4^+^ T	C57BL/6 mouse, Human	EAU, sympathetic ophthalmia	Enhanced Th17 development and pathogenicity by blunting the suppressive effect of miR-182 on TAF15/STAT3 pathway	(51)
miR-182, miR-96, miR-183	Down	Ocular tissue	B10.RIII mouse	EAU	N/A	(91)

EAAU, experimental autoimmune anterior uveitis; EAE, experimental autoimmune encephalomyelitis; EAU, experimental autoimmune uveitis/uveoretinitis; LN, lupus nephritis; RA, rheumatoid arthritis; RRMS, relapse remitting multiple sclerosis; SLE, systemic lupus erythematosus. N/A, Not Assessed.

### 4.1 miR-183C in SLE

The overexpression of miR-183C miRNAs has been identified in splenic lymphocytes of different lupus-prone mice models, including MRL/lpr, C57BL/6-lpr, NZB/W_F1_, and C3.MRL-Fas^lpr^, and in peripheral blood mononuclear cells (PBMCs) of MRL/lpr mice (**Table 1**) (28, 79, 80). The upregulation of miR-182 has also been reported in PBMCs of human lupus patients (82). Inhibition of miR-182 alone or three miR-183C miRNAs simultaneously *in vitro* in splenocytes of MRL/lpr mice significantly inhibited either LPS or Concanavalin A (Con A) induced production of proinflammatory cytokines IFNγ and IL-6 (34). These *in vitro* data suggested a proinflammatory role of miR-182 and miR-183C in lupus. To further understand the effect of miR-182 and miR-183C miRNAs *in vivo* on lupus, we developed B6/lpr models with conditional deletion of miR-182 alone (miR-182^-/-^B6/lpr) or whole miR-183C cluster (miR-183C^-/-^B6/lpr) in CD2^+^ lymphocytes. Deletion of miR-182 alone or miR-183C in B6/lpr mice did not exhibit an obvious effect on body and spleen weight, proteinuria level, and renal histopathological score but significantly inhibited IgG deposition in kidneys. However, in miR-183C^-/-^B6/lpr mice, the production level of anti-dsDNA autoantibodies was significantly reduced in a time-dependent manner, suggesting the ameliorating effect of miR-183C deletion on lupus *in vivo* (34). Consistently, there was a significant reduction of IFNγ production in *in vitro* stimulated splenocytes from either miR-182^-/-^B6/lpr or miR-183C^-/-^B6/lpr mice compared to their respective controls. Further, we demonstrated that miR-183C regulated IFNγ production in splenocytes by targeting Foxo1.

The ameliorative effect of miR-182 inhibition on lupus has also been reported by Wang et al. (81). In this study, increased miR-182 expression in high Chronicity Index (CI) LN patients and in lupus-prone MRL/lpr mice was associated with the development of LN and reduced Foxo1 expression. *In vivo* inhibition of miR-182 in MRL/lpr mice with antagomir-182 delayed LN progression with attenuation of tissue damage and improved renal functions (81). Transforming growth factor-β (TGF-β) signaling plays a major role in driving tissue fibrosis, a pathological process during LN development (92, 93). The authors further demonstrated that TGF-β1 treatment promoted miR-182 expression in human renal tubular epithelial HK-2 cells and mouse glomerular mesangial SV40 MES 13 cells, suggesting a potential involvement of miR-182 in renal fibrosis during LN development.

Nevertheless, reduced miR-183 expression was identified in renal tissues of LN patients and lupus mice, which suggested a protective effect of miR-183 on LN (84, 85). One group reported that intraperitoneal injection of miR-183 mimic into MRL/lpr mice led to a significant reduction in serum anti-dsDNA levels, blood urea nitrogen (BUN) and urinary albumin levels (84). The MRL/lpr mice injected with miR-183 mimic also had reduced immunocomplex (IgG and C3) deposition in the kidneys and a prolonged survival rate compared to the mice receiving the control miRNA mimic (84). The authors further demonstrated that miR-183 exhibited a protective effect on lupus *via* targeting mammalian target of rapamycin (mTOR) since there was reduced mTOR expression and activation in miR-183 mimic-treated cells. The ameliorating effect of mTOR inhibition on lupus has been well documented (94, 95). Another study reported that overexpression of miR-183 in MRL/lpr mice *via* caudal vein injection of miR-183 agomiR reduced renal injury with decreased renal histopathological scores, reduced expression of proinflammatory cytokines (IL-6 and TNF-α) and renal fibrosis-related factors (α-SMA and Vimentin) in the kidney (85). The inhibition of miR-183 *via* injecting the mice with antagomir-183 had opposite effects. Moreover, the inhibitory effect of miR-183 on proinflammatory cytokines and renal fibrosis-related factors was verified in human renal glomerular endothelial cells. Mechanistically, Qi et al. demonstrated that miR-183 attenuated LN by targeting transforming growth factor beta receptor 1 (Tgfbr1), an enhancer of the TGF-β/Smad/TLR3 pathway (85).

In summary, miR-182 and miR-183 demonstrated tissue-specific dysregulation in lupus and exhibited opposite roles on LN. It is noteworthy that a single base difference between seed sequences of miR-183 and miR-182 gives them distinct mRNA target genes. Thus, further investigations about the specific and overlapping target genes of individual miR-183C miRNAs under an SLE background would assist us in illuminating the similar and divergent roles of miR-183C miRNAs in autoimmunity and autoinflammation.

### 4.2 miR-183C in MS and EAE

The abnormal expression of miR-183C miRNAs has been identified in the immune cells of MS patients and the murine model of MS, EAE mice (**Table 1**) (27, 29). Several groups have evaluated the pathogenic role of miR-183C miRNAs in EAE. It has been reported that miR-182 was upregulated in a Chinese cohort of patients with relapsing and remitting multiple sclerosis (RRMS), which is associated with the increase of IFNγ-expressing Th1 cells (27). The *in vivo* study indicated that transgenic overexpression of miR-182 in mice promoted MOG_35-55_-induced EAE with increased frequency of Th17 and Th1 cells, early onset of the disease, and severe symptoms. In contrast, the EAE mice with global knockdown of miR-182 showed mitigated disease symptoms with a significantly lower frequency of Th17 and Th1 cells compared to controls (27). The promoting effect of miR-96 and miR-183C on the onset of EAE in mice has been reported (33). miR-183C miRNAs, particularly miR-96, drove the Th17 pathogenicity in autoimmune disease. Compared to controls, the mice receiving miR-96-transduced MOG-specific TCR transgenic (2D2) Th17 cells developed much more severe EAE, accompanied by a higher frequency of IL-17 and GM-CSF expressing cells in the central nervous system. In addition, the authors showed that the *RAG1* ^-/-^ mice that received CD4^+^ T cells from miR-183C^-/-^ mice developed less severe EAE with reduced frequency of IL-17 and GM-CSF expressing T cells when compared to the *RAG1* ^-/-^ mice received CD4^+^ T cells from miR183C^+/+^ mice (33). These studies strongly suggested that miR-183C miRNAs have a promoting effect on the pathogenesis of MS and EAE by driving the pathogenicity of Th17 cells, a major pathogenic contributor in MS and EAE autoimmune disease.

### 4.3 miR-183C in autoimmune-mediated eye diseases

miR-183C miRNAs are critical for retinal development and function. Dysregulated miR-183C expression has also been implicated in the pathogenesis of autoimmune-mediated eye diseases such as autoimmune uveitis and Grave’s orbitopathy (GO) (**Table 1**) (49, 51, 96). miR-183C miRNAs were downregulated in the ocular tissues of EAU mice, correlating with the upregulation of IL-17 in the eye (91). The downregulation of miR-182 was also detected in CD4^+^ T cells of EAU mice and in the ocular tissues and CD4^+^ T cells of human patients with sympathetic ophthalmia (SO) (51, 90). Zhang et al. further demonstrated that miR-182 negatively regulated Th17 development and pathogenicity in EAU *via* targeting TAF15 (51). The mice receiving miR-182 mimic transfected Th17 cells had ameliorated clinical EAU scores compared to the mice receiving control mimic transfected Th17 cells. This study suggested a therapy role of miR-182 mimic in the EAU (51).

In contrast to the downregulation of miR-182 in EAU mice and human patients with SO, miR-182 and miR-183 were upregulated in ocular tissues of experimental autoimmune anterior uveitis (EAAU) rat (89), and miR-96 and miR-183 were elevated in CD4^+^ T cells of human patients with GO (49). Elevated miR-96 and miR-183 expression contributed to enhanced T cell proliferation and activation by targeting EGR1/PTEN/Akt pathway (49). The genetic variant of miR-182 (rs76481776 CC→TT/CT) was associated with the susceptibility of human Vogt-Koyanagi-Harada (VKH) syndrome and Behçet’s disease, which are characterized by bilateral granulomatous and non-granulomatous uveitis, respectively (97). Further, Yu et al. demonstrated that miR-182 expression was significantly increased in the activated CD4^+^ T cells from healthy controls with rs76481776 TT/CT genotype compared to rs76481776 CC genotypes (97).

## 5 Conclusions and perspectives

This review summarizes the complex role of miR-183C miRNAs, either individually or the whole cluster, in different types of immune cells and autoimmune diseases. Most, but not all, studies found that miR-183C miRNAs are proinflammatory and upregulated in autoimmune disorders, especially in SLE and MS/EAE (**Table 1**). These findings suggest that miR-183C miRNAs can potentially be diagnostic biomarkers and/or therapeutic targets for specific autoimmune diseases.

The therapeutic effects of inhibiting miR-183C miRNAs *in vivo* in animal models of autoimmune diseases (such as SLE and MS) have been confirmed by different research groups. In these studies, inhibition or deletion of miR-183C whole cluster or individual miR-183C miRNA *in vivo* led to the attenuation of disease symptoms in lupus mice and in EAE mice (27, 33, 34, 81). These data are highly promising for developing miR-183C miRNAs-based therapy for specific autoimmune disorders treatment. Given the importance of miRNAs in biological functions and the pathogenesis of different diseases, a number of miRNA-based therapeutic approaches are being developed and entered into clinical trials. The major challenges to miRNA-based therapy include developing efficient and targeted delivery systems, dosage concerns, degradation caused by nucleases, and off-target effects (98, 99). ABX464, which upregulates miR-124, was approved for clinical trials to treat ulcerative colitis and rheumatoid arthritis (100–102). RG-012, an effective inhibitor of miR-21, is undergoing Phase 2 HERA trials for Alport syndrome; and MRG-201, the agonist to miR-29b, is in Phase 1 clinical trial for scleroderma (103, 104). These clinical trials may eventually pave the way for using other disease-specific miRNAs in autoimmune and inflammatory diseases.

Since miR-183C miRNAs are involved in embryonic development and regulation of various biological functions of other organs, it is imperative that miR-183C-based treatment requires achieving stable and cell-specific delivery to minimize potential unforeseen side effects. In general, the miR-183C miRNAs studies have been shown to regulate a broad range of immunological pathways, including the development, differentiation, and cytokine production of various immune cells. Each miRNA in miR-183C has both overlapping redundant and unique targets due to minimal differences in seed sequences. This allows miR-183C miRNAs to target gene expression cooperatively or individually. miR-183C’s regulatory effects on the immune system or inflammatory diseases should not be generalized and need to be evaluated in the specific disease context as they were differentially dysregulated in different types of autoimmune diseases (**Table 1**). Thorough investigations of miR-183C miRNAs, both individually and collectively, in cell-specific and disease-specific contexts are essential prerequisites for developing innovative diagnostic and therapeutic alternatives.

## Author contributions

ZW drafted and wrote the manuscript. RD assisted with the writing and critically reviewed and edited the manuscript. SA critically reviewed and edited the manuscript. All authors contributed to the article and approved the submitted version.
